# Protective impacts of bamboo leaf flavonoids in stressed broilers induced by diquat: Insight of antioxidant, immune response and intestinal barrier function

**DOI:** 10.1016/j.aninu.2024.11.001

**Published:** 2024-11-20

**Authors:** Qilu Zhou, Sikandar Ali, Xueyan Shi, Guangtian Cao, Jie Feng, Caimei Yang, Ruiqiang Zhang

**Affiliations:** a College of Animal Science and Technology, College of Veterinary Medicine, Zhejiang Agricultural and Forestry University, Hangzhou 311300, Zhejiang, China; b Zhejiang Vegamax Biotechnology Co., Ltd., Anji 313300, Zhejiang, China; c College of Quality and Standardization, China Jiliang University, Hangzhou 310018, Zhejiang, China; d College of Animal Science, Zhejiang University, Hangzhou 310058, Zhejiang, China

**Keywords:** Bamboo leaf flavonoid, Growth performance, Antioxidant, Intestinal barrier function, Broiler, Diquat

## Abstract

This research explored the protective impact of bamboo leaf flavonoids (BLF) in diquat (DQ) stressed broilers; providing insight of antioxidant, immune response and intestinal barrier function. This experiment consisted of two parts. In the first, 240 chicks were allotted to 2 groups with 8 replicates and 15 chicks per replicate. Treatments consisted of a basic feed (control group, CON) and the basic feed plus 1000 mg/kg BLF (BLF group, BLF) for 28 d, respectively. Then, following the conclusion of the first part, 16 healthy broilers were selected from the CON group and the BLF group. They formed the second part of the experiment, and were allotted to 4 treatments with 8 broilers each: CON-no stress (CON-NS) group, CON-DQ group, BLF-NS group and the BLF-DQ group. Broilers were separately injected intraperitoneally with DQ solution at 40 mg/kg body weight or the same dose of phosphate buffer saline. The results revealed adding BLF to diet reduced the ratio of feed to weight gain of broilers compared to the basic feed group (*P* = 0.021). In comparison to the CON-NS group, BLF improved the levels of serum and jejunal mucosa total antioxidant capacity, immunoglobulin M, serum catalase, immunoglobulin A, interleukin 10, jejunal mucosa interleukin 4, cecal butyric acid, valeric acid, isobutyric acid, isovaleric acid, upregulated zonula occludens-1 (*ZO-1*), occludin (*OCLN*) and claudin-1 (*CLDN1*) expressions, and reduced the levels of jejunal mucosa malondialdehyde (MDA), interleukin 1β, interleukin 6 and serum diamine oxidase (*P* < 0.05). Diquat stress elevated the contents of serum MDA, D-lactate, jejunal mucosa tumor necrosis factor α, reactive oxygen species and *unclassified_f_Lachnospiraceae* relative abundance, downregulated *ZO-1*, *OCLN* and *CLDN1* expressions, and reduced Sobs, Chao and Ace indices (*P* < 0.05). Compared with CON-NS group, the concentration of isovaleric acid in the BLF-DQ group was higher (*P* < 0.05). In conclusion, by establishing a DQ stress injury model, it was elucidated that BLF may enhance antioxidant capacity, strengthen immunity, regulate volatile fatty acid contents, improve intestinal morphology, microbiota and other intestinal barrier functions, so as to mitigate the injury induced by oxidative stress in broilers.

## 1 Introduction

Broilers possess the characteristics of high protein and low fat, which are in great demand in the world (Nasr et al., 2021). With the increase in consumption of broilers, the poultry industry has developed rapidly, showing an intensive trend, but it has also triggered a series of stress reactions and posed threats to the health of poultry (Song et al., 2024). The excessive production of free radicals and the damage to antioxidant defenses are the main factors causing harmful consequences from poultry oxidative stress (Surai et al., 2019). The intestine, an essential organ for food digestion and nutrient absorption, is one of the largest mucosal regions in the body. A convection oxygen delivery mechanism makes it more vulnerable to oxidative stress damage, resulting in an imbalance of intestinal flora (Garza-Lombó et al., 2020; Xiao et al., 2017). Oxidative stress may cause abnormal immune responses and intensified inflammatory reactions in broilers, thereby affecting their health and growth performance, and resulting in considerable losses to the development of the poultry industry (Hu et al., 2023). Therefore, finding suitable and healthy natural antioxidants is crucial for the poultry industry.

Bamboo extracts contain abundant flavonoids and other active substances, which have excellent antioxidant activity and could be used as readily available and valuable biological sources of natural antioxidants (Gong et al., 2015). An *in vivo* study has shown that flavonoids in bamboo leaf extracts exhibit anti-inflammatory effects at higher doses and anti-angiogenic activity at lower concentrations (Wedler et al., 2014). Bamboo leaf flavonoids (BLF), a natural organic mixture, are the main functional factor in bamboo leaf extracts which exist obvious antioxidant, anti-inflammatory, lipid regulating, anti-tumor and other pharmacological activities (Kimura et al., 2022; Ma et al., 2020). Bamboo leaf flavonoids have notable free radical scavenging ability and could initiate antioxidant defense response by increasing the expression of related signaling pathways (Nrf2/HO-1/NQO1) to alleviate cellular oxidative stress (Yu et al., 2019). Previous study has confirmed that BLF contribute to inhibiting the polarization and activation of macrophages and dendritic cells, reducing the production of inflammatory mediators (Yang et al., 2017). Furthermore, research has found that dietary supplementation with 100 or 400 mg/kg bilberry extracts - water-soluble flavonoids - could increase the activity of antioxidant enzymes in the liver, jejunal mucosa, and plasma in yellow feathered broilers (Wang et al., 2021b). Nevertheless, more studies are required to explore the impact of BLF to alleviate oxidative stress and regulate the inflammatory response and intestinal barrier function of yellow feathered broilers.

Diquat (DQ), a commonly used bipyridyl herbicide, generates a large amount of superoxide anion free radicals, which attacks mitochondria and creates excessive reactive oxygen species (ROS), causing oxidative damage to animals (Nisar et al., 2015). Animal intraperitoneal injection of DQ is often used as a chemical model for studying animal oxidative stress and has a high reference value (Koch and Hill, 2017). In poultry, researchers have established an oxidative stress model of liver injury in broilers by injecting 20 mg/kg body weight (BW) kg DQ solution into the abdominal cavity (Chen et al., 2021b). In mammals, it has been discovered that DQ stress altered intestinal permeability in piglets, damaged mitochondrial function and led to mitochondrial autophagy (Cao et al., 2018).

Consequently, this research intends to discover the protective impacts of BLF on growth performance, antioxidants, immune response, and intestinal barrier function in stressed broilers induced by DQ so as to provide effective measures and theoretical references for repairing oxidative stress injury in animals.

## 2 Materials and methods

### 2.1 Animal ethics statement

All steps of the animal experiment were approved by the Research Center Institutional about Animal Care and Use Committee of Zhejiang Agricultural and Forestry University (ZAFUFB2022031).

### 2.2 Experimental design and broilers management

This experiment consisted of two parts. The first part was the study of the impacts of BLF on the growth performance of chicks. Two hundred and forty fast growth yellow feathered chicks at 1-d-old were selected and stochastically allotted to 2 treatments (average initial body weight = 29.99 ± 0.421 g) with 8 replicates and 15 chicks in each, with each replicate being assigned to an identical cage (length 2.0 m × width 0.85 m × height 0.6 m) for feeding. The broilers in control group (CON) were raised with a basic feed, while the broilers in BLF group were raised with the basic feed added with BLF at 1000 mg/kg. The dose selection referred to the standards of the prevenient study (Wu et al., 2024). The bamboo (*Dendrocalamus membranaceus*) leaf flavonoids were supplied by Zhejiang Vegamax Biotechnology Co., Ltd. (Anji, China) (Cao et al., 2022). They mainly composed of 42.5% flavonoids, 8.3% lactones and 9.4% phenolic acids. All broilers were raised under the same light conditions (23 h of light and 1 h of dark per day) and allowed to feed and drink freely for 28 d. Temperature, humidity and vaccination were controlled according to the requirements of broiler breeding regulations. The initial weight and feed consumption of broilers during the experiment were recorded. After fasting for 12 h, the broilers were weighed at the age of 28 d.

The second part was an exploration of the protective effects of BLF in broilers under DQ stress. At the end of the first part of the experiment, 16 healthy 28-d-old broilers were respectively selected from the CON group and BLF group, and assigned to 4 groups with 8 broilers in each: CON-no stress (CON-NS), CON-DQ, BLF-DQ and BLF-NS. The broilers in CON-NS group and CON-DQ group were from the basic diet feeding group. The broilers in BLF-NS group and BLF-DQ group were from the BLF group of the first part of the experiment. At 29 d of age, broilers were separately injected intraperitoneally with DQ solution at a dosage of 40 mg/kg of BW as in previous studies (Chen et al., 2021b) or the same dose of phosphate buffer saline (PBS) at 08:00. The DQ was bought from Sigma–Aldrich Corporation (St. Louis, USA), and 40 mg/mL DQ solution and PBS were prepared before injection. Samples were taken 24 h after injection DQ or PBS solution.

### 2.3 Diets and analyses

The composition and nutritional level in this work are shown in Table 1. The preparation of basic feed was according to the Nutrient Requirements for Yellow Feather Broilers (NY/T 3645-2020) (Ministry of Agriculture of the People’s Republic of China, 2020). The contents of crude protein (CP) in the feed were determined by the Kjeldahl method according to China National Standard (GB/T 6432-2018). The contents of calcium (Ca) were determined by EDTA disodium complexometric titration according to China National Standard (GB/T 6436-2018). The contents of total phosphorus (TP) were determined by spectrophotometry according to China National Standard (GB/T 6437-2018). The metabolizable energy level and amino acid content were calculated using the method of the previous study (Kong and Adeola, 2014) and data provided by China Feed Database (2021) and the proportion of each raw material in the formula.

**Table 1:** Composition and nutrient levels of basal diet (air-dry basis, %).

Item	Content
**Ingredients**	
Corn	53.00
44% soybean meal	24.50
Expanded soybean	5.00
Corn distiller’s grains	8.00
Soya oil	1.70
Mineral meal	1.30
Fermented soybean meal	2.50
Premix^1^	4.00
Total	100.00
**Nutrient levels**^2^	
ME, kcal/kg	2916.00
CP	20.30
Lys	1.19
Met + Cys	0.89
Ca	0.87
TP	0.60

ME = metabolizable energy; CP = crude protein; Ca = calcium; TP = total phosphorus.

1 The premix provided the following per kilogram of diets: vitamin A 10,000 IU, vitamin D_3_ 2000 IU, vitamin E 30 IU, vitamin K 35 g, vitamin B_1_ 1.5 mg, vitamin B_2_ 3.5 mg, vitamin B_6_ 3 mg, vitamin B_12_ 10 μg, D-pantothenic acid, 10 mg, nicotinic acid 30 mg, biotin, 0.15 mg, choline chloride 1000 mg, Fe (as ferrous sulfate) 80 mg, Cu (as copper sulfate) 8 mg, Mn (as manganese sulfate) 60 mg, Zn (as zinc sulfate) 40 mg, Se (as sodium selenite) 0.15 mg, I (as potassium iodide) 0.18 mg.

2 Metabolizable energy and amino acid were calculated values, while CP, Ca and TP were measured values.

### 2.4 Samples collection

After 24 h of injection of DQ solution or PBS, no mortality of broilers was observed, and 8 samples were taken respectively from CON-NS, CON-DQ, BLF-NS, and BLF-DQ groups. The blood samples were collected under the wings and placed at room temperature for 30 min. The serum samples were stored after the blood samples were centrifuged at 4450×*g* for 15 min at 4 °C. Then, the broilers were euthanized and dissected to obtain small intestine samples. The intestinal chyme samples were rinsed with physiological saline. About 1.5 cm of the middle of the jejunal segments were sliced, preserved and fixed. The intestinal segment was kept in 4% paraformaldehyde solution for morphological analysis. The mucosa was scraped from the posterior jejunum, and the chyme was collected from the cecum. The cecal chyme samples were aseptically cut and immediately moved to sterile low-temperature vials and frozen until later determinations of volatile fatty acid (VFA) and cecal microflora. The above serum and intestinal samples were preserved at −80 °C.

### 2.5 Serum and jejunal mucosa related indicators

An appropriate amount of small intestinal mucosa sample was added with precooled normal saline in the ratio of 1:4 (g/mL), ground in an ice environment, and centrifuged for 10 min at 3500 × *g*. The supernatants of intestinal homogenate and serum were taken to determine the following indicators: D-lactate (D-LA; Cat. No. A019-3-1), diamine oxidase (DAO; Cat. No. A088-1-1), reactive oxygen species (ROS; Cat. No. E004-1-1), total antioxidant capacity (T-AOC; Cat. No. A015-2-1), catalase (CAT; Cat. No. A007-1-1), glutathione peroxidase (GPx; Cat. No. H545-1-1), total superoxide dismutase (T-SOD; Cat. No. A001-3-2) and malondialdehyde (MDA; Cat. No. A003-1-2). The above reagent kits were bought from Nanjing Jiancheng Biotechnology Co., Ltd. (Nanjing, China), and the specific operation processes were strictly followed according to the instructions.

ELISA kits (Nanjing Angle Gene Bioengineering Co., Ltd., Nanjing, China) were used to detect serum and intestinal mucosal immunoglobulins and cytokine related indicators, including immunoglobulin A (IgA; Cat. No. ANG-E32004C), secretory immunoglobulin A (sIgA; Cat. No. ANG-E32006C), immunoglobulin Y (IgY; Cat. No. ANG-E32209C), immunoglobulin M (IgM; Cat. No. ANG-E32005C), interleukin 4 (IL-4; Cat. No. ANG-E32064C), interleukin 10 (IL-10; Cat. No. ANG-E32011C), interleukin 1β (IL-1β; Cat. No. ANG-E32031C), tumor necrosis factor α (TNF-α; Cat. No. ANG-E32030C) and interleukin 6 (IL-6; Cat. No. ANG-E32013C). The ELISA kits were only used to determine the relevant indicators of broiler samples, and the coefficients of variation within and between batches were, respectively, less than 9% and 15%.

### 2.6 Intestinal histomorphology

The samples of jejunum were fixed in 4% formaldehyde solution for 24 h, dehydrated in gradient concentration ethanolic solution, washed with xylene and embedded in paraffin. The microtome (KH-Q320, Hubei Xiaogan Kuaohai Medical Technology Co., Ltd., Hubei, China) was used to cut fixed samples into 5 μm thickness. After staining the slices, the distance from the tip of villus to the crypt opening (villus height) and distance from the crypt opening to the base (crypt depth) were measured under a microscope (Eclipse ci, Nikon Precision Shanghai Co., Ltd., Shanghai, China). Six representative and complete visual fields were selected from each intestinal cross section for photography and measurement.

### 2.7 Intestinal genes expressions

Total RNA was extracted from jejunal mucosa with Trizol total RNA Extraction Kit (Takara Biomedical Technology Co., Ltd., Beijing, China) and Nano-300 spectrophotometer (Hangzhou Allsheng Instruments Co., Ltd., Hangzhou, China) was used to determine the content. The target gene was synthesized by Tsingke Biotech Technology Co., Ltd. (Hangzhou, China) and the primer sequence is presented in Table 2. The PCR program was pre-denatured at 95 °C for 5 min, denatured at 95 °C for 30 s, annealed at 60 °C for 30 s, extended at 72 °C for 30 s. The fold expression of each gene was determined using the 2^−ΔΔCt^ method, with the β-actin gene serving as an internal standard.

**Table 2:** Primer sequence of fluorescent quantitative PCR.

Gene	Primer sequence (5′→3′)	GenBank number
*ZO*-1	F: AGCGAAGCCACCTGAAGATA R: GATGGCCAGCAGGAATATGT	XM_032893463.1
*OCLN*	F: AGTACATGGCTGCTGCTGATG R: CCCACCATCCTCTTGATGTGT	XM_032898729.1
*CLDN1*	F: CTGGGAGGTGCCCTACTTT R: CCGCTGTCACACGTAGTCTT	XM_032900042.1
β-Actin	F: GATTACTGCCCTGGCTCCTA R: TCATCGTACTCCTGCTTGCT	NM_031144

*ZO-1* = zonula occludens-1; *OCLN =* occluding; *CLDN1* = claudin-1.

### 2.8 Volatile fatty acids in cecal contents

The cecal chyme sample was accurately weighed to 1 g and blended with 3 mL 6% phosphoric acid and took supernatant after centrifugation at 12,000 × *g* for 10 min at 4 °C and separated the supernatant to 25% (wt/vol, 1:5) metaphosphate. After an ice bath time of more than 30 min, the sample was centrifuged at 12,000 × *g* for 10 min at 4 °C again. A gas chromatograph (GC7890B, Agilent Technologies, California, USA) furnished with a 30 mm × 0.53 mm inner diameter column (Teknokroma TRB-FFAP, Barcelona, Spain) and a flame ionization was used to detect the sample. After comparing peak areas of the standard solution and the sample, the VFA content in each sample was calculated by integration.

### 2.9 Microbial flora of cecal contents

The DNA extraction kit (Qiagen GmbH, Hilden, Germany) was used to extract microbial DNA from each intestinal chyme sample. The purity and concentration of genomic DNA were evaluated by 1% agarose gel electrophoresis. PCR amplification of the 16S rRNA gene was performed using forward primers 5′ - ACCTACGGGGCAG - 3′ and reverse primers 5′ - GACTACVGGGTWTCTAAT - 3′. The libraries of purified PCR products were sequenced using NEXTFLEX Rapid DNA Seq Kit. Sequencing and related biological analysis were conducted on NovaSeq PE 300 platform (Illumina, USA). The sequenced samples were split, the paired-end reads were filtered according to the sequencing quality, and the optimized data were obtained by splicing according to the overlapping relationship. The optimized data were processed by Divisive Amplicon Denoising Algorithm 2, and amplicon sequence variant (ASV) was obtained to represent the sequence and abundance information for visual analysis. The gene sequence data was analyzed using UPARSE (7.1 version), chimeric filtering, species annotation, and abundance analysis. The microbial community composition was analyzed based on tax summary and R software package version 3.3.1.

### 2.10 Statistical analysis

Excel 2019 was used to preliminarily sort out data, and SPSS 21.0 software (SPSS Inc., USA) was used for further data analysis. The growth performance related indicators were analyzed by independent-sample T test. The data of the remaining indicators were analysed using Gaussian linear mixed model with SPSS for all variables.

$$Y_{ijk}=\mu +F_{i}+I_{j}+R_{k}+e_{ijk}$$

where $Y_{ijk}$ is the dependent variable; μ, the overall mean; $F_{i}$, the i th fixed effect of the feed (*n* = 2); $I_{j}$, the j th fix effect of injection (*n* = 2); $R_{k}$, the k th random effect of individuals (*n* = 32), $e_{ijk}$, the residual error. Results were presented as the least squares means ± SEM. The 5% level of statistical significance (*P* < 0.05) was used as the threshold. The GraphPad Prism 8 (GraphPad Prism Inc., USA) was used for drawing.

## 3 Results

### 3.1 Growth performance

Impacts of BLF on growth performance in broilers are presented in Table 3. The results indicated that adding BLF to feed could significantly reduce the ratio of feed to weight gain (F/G) in broilers when compared to CON group (*P* = 0.021).

**Table 3:** Effects of the bamboo leaf flavonoids on growth performance in broilers (1-28 d of age).

Item	CON group	BLF group	*P*-value
Final body weight, g	669.51 ± 39.158	678.02 ± 29.127	0.629
Average daily feed intake, g	40.21 ± 2.712	39.01 ± 2.879	0.988
Average daily gain, g	22.60 ± 1.421	22.98 ± 1.041	0.553
Ratio of feed to weight gain	1.78 ± 0.043	1.69 ± 0.083	0.021

### 3.2 Serum and jejunal mucosa antioxidant indicators

**Fig. 1:**
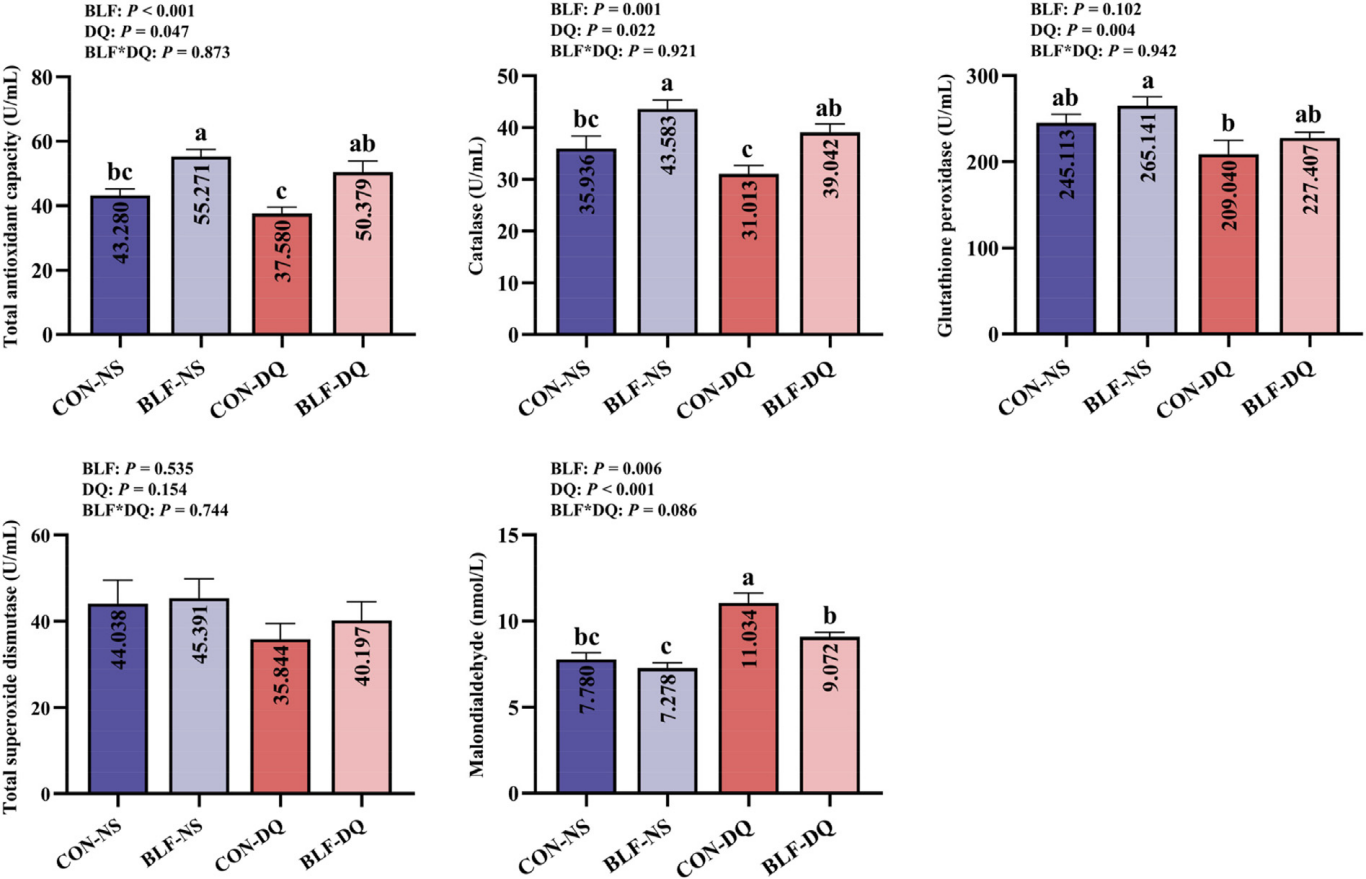
Effects of the bamboo leaf flavonoids (BLF) on serum antioxidant parameters in diquat (DQ) stressed broilers. Columns labeled with different letters indicate significant differences (*P* < 0.05), while with no letter or the same letter superscripts mean no significant difference (*P* > 0.05). *n* = 8.

Effects of the BLF in serum antioxidant parameters in broilers under DQ stress are shown in Fig. 1. Two-way ANOVA test displayed that dietary supplementation with BLF improved serum T-AOC (*P* < 0.001) and CAT activities (*P* = 0.001), decreased MDA content (*P* = 0.006). Diquat stress diminished the levels of T-AOC (*P* = 0.047), CAT (*P* = 0.022), GPx (*P* = 0.004), augmented MDA content (*P* < 0.001). In comparison to CON-NS group, BLF-NS group improved the serum T-AOC level and CAT activity (*P* < 0.05), and added serum MDA content in CON-DQ group (*P* < 0.05). Compared with CON-DQ group, the contents of serum T-AOC and CAT were increased (*P* < 0.05), the MDA level was reduced in BLF-DQ group (*P* < 0.05). No significant discrepancies in serum T-AOC, CAT, GPx, T-SOD, MDA contents between CON-NS group and BLF-DQ group of broilers were observed (*P* > 0.05).

**Fig. 2:**
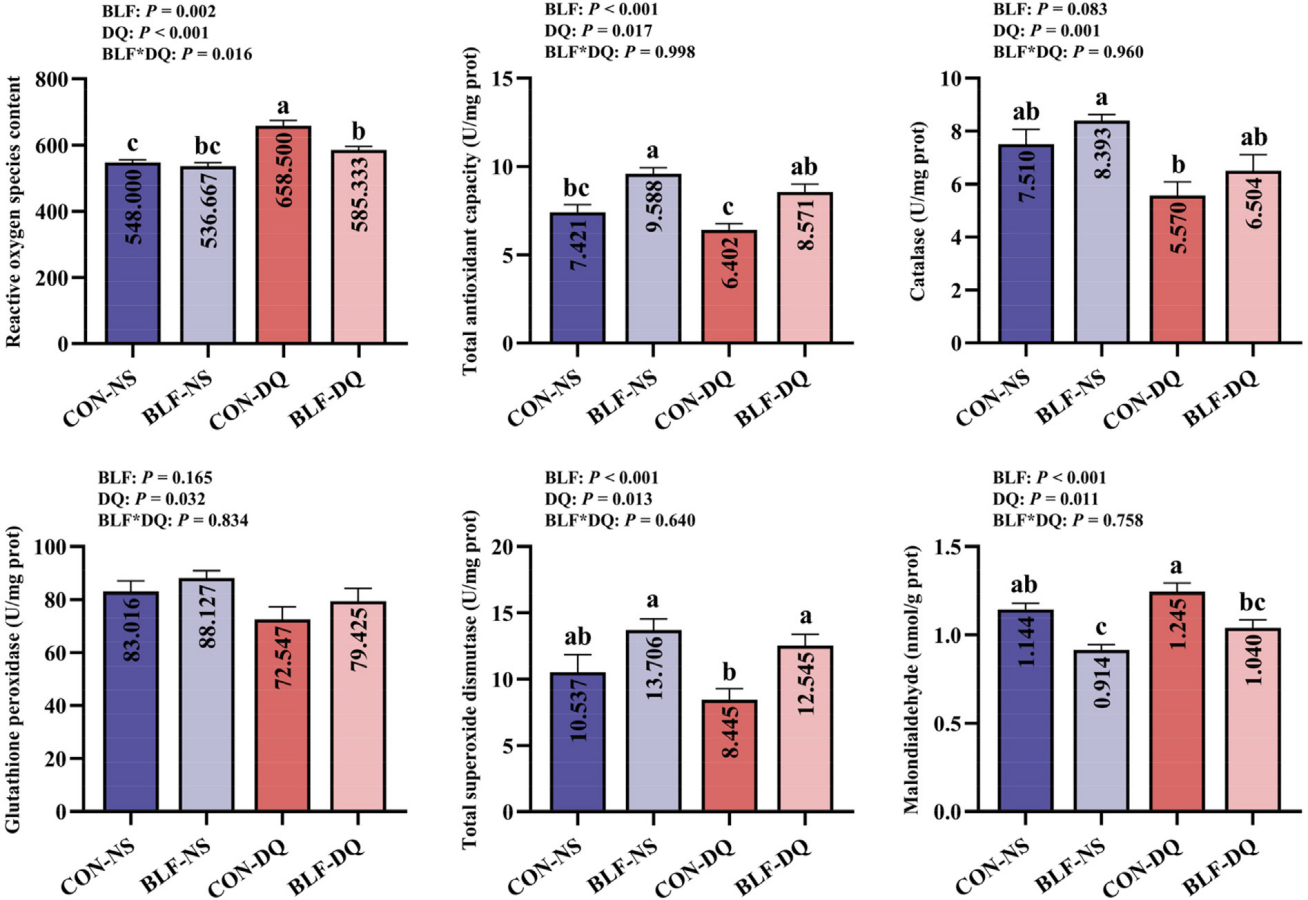
Effects of the bamboo leaf flavonoids (BLF) on jejunal mucosa antioxidant parameters in diquat (DQ) stressed broilers. Columns labeled with different letters indicate significant differences (*P* < 0.05), while with no letter or the same letter superscripts mean no significant difference (*P* > 0.05). *n* = 8.

Adding BLF to diet reduced jejunal mucosa ROS (*P* = 0.002) and MDA levels (*P* < 0.001), enhanced T-AOC (*P* < 0.001) and T-SOD contents (*P* = 0.001; Fig. 2). Diquat stress increased ROS (*P* < 0.001) and MDA (*P* = 0.011) concentrations, declined the levels of jejunal mucosa T-AOC (*P* = 0.017), CAT (*P* = 0.001) and GPx (*P* = 0.032). There was an interactive effect of supplementing BLF and DQ stress on ROS activity in the jejunal mucosa (*P* = 0.016). Broilers in BLF-DQ group had lower contents of jejunal mucosa ROS and MDA (*P* < 0.05), higher levels of T-AOC and T-SOD (*P* < 0.05) when compared to the CON-DQ group. Results indicated no significant differences in the levels of jejunal mucosa T-AOC, CAT, GPx, T-SOD, and MDA between CON-NS group and BLF-DQ group of broilers (*P* > 0.05).

### 3.3 Serum D-LA and DAO

**Fig. 3:**
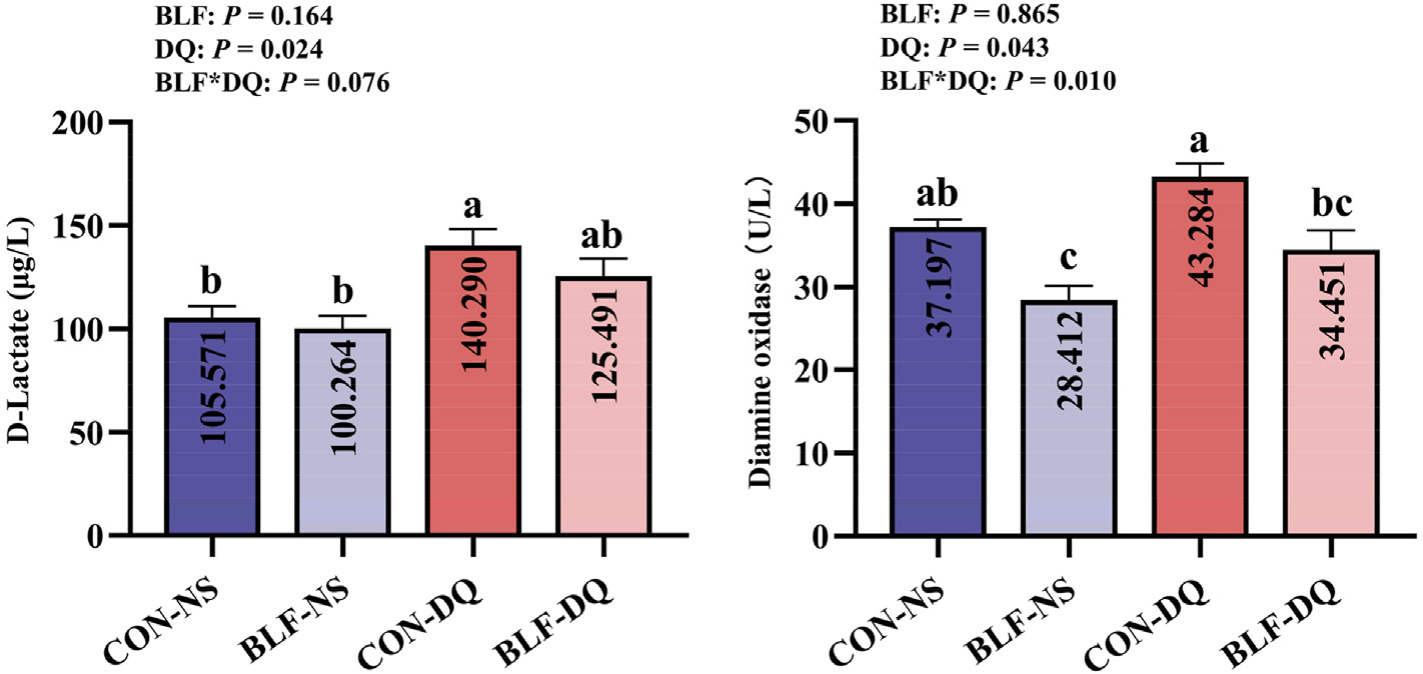
Effects of bamboo leaf flavonoids (BLF) on serum D-lactate and diamine oxidase in serum in diquat (DQ) stressed broilers. Columns labeled with different letters indicate significant differences (*P* < 0.05), while with no letter or the same letter superscripts mean no significant difference (*P* > 0.05). *n* = 8.

Diquat stress significantly improved serum D-LA (*P* = 0.024) and DAO (*P* = 0.043) levels, and the addition of BLF and DQ stress had an interaction on DAO activity (*P* = 0.010; Fig. 3). Compared with CON-NS group, the serum DAO activity in BLF-NS group was cut down (*P* < 0.05), the D-LA level in CON-DQ group was scaled up (*P* < 0.05). The serum DAO of broilers in BLF-DQ group was reduced compared to CON-DQ group (*P* < 0.05). The differences between CON-NS group and BLF-DQ group in serum D-LA and DAO were not significant (*P* > 0.05).

### 3.4 Serum and jejunal mucosa immunoglobulins

**Fig. 4:**
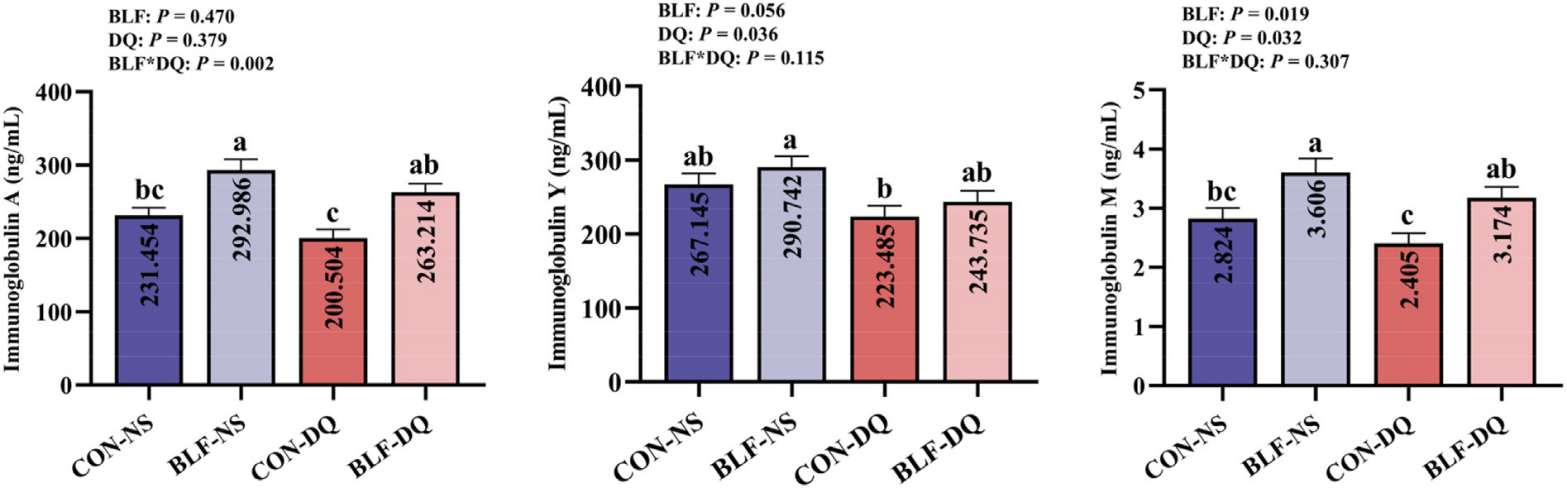
Effects of the bamboo leaf flavonoids (BLF) on serum immunoglobulins in diquat (DQ) stressed broilers. Columns labeled with different letters indicate significant differences (*P* < 0.05), while with no letter or the same letter superscripts mean no significant difference (*P* > 0.05). *n* = 8.

**Fig. 5:**
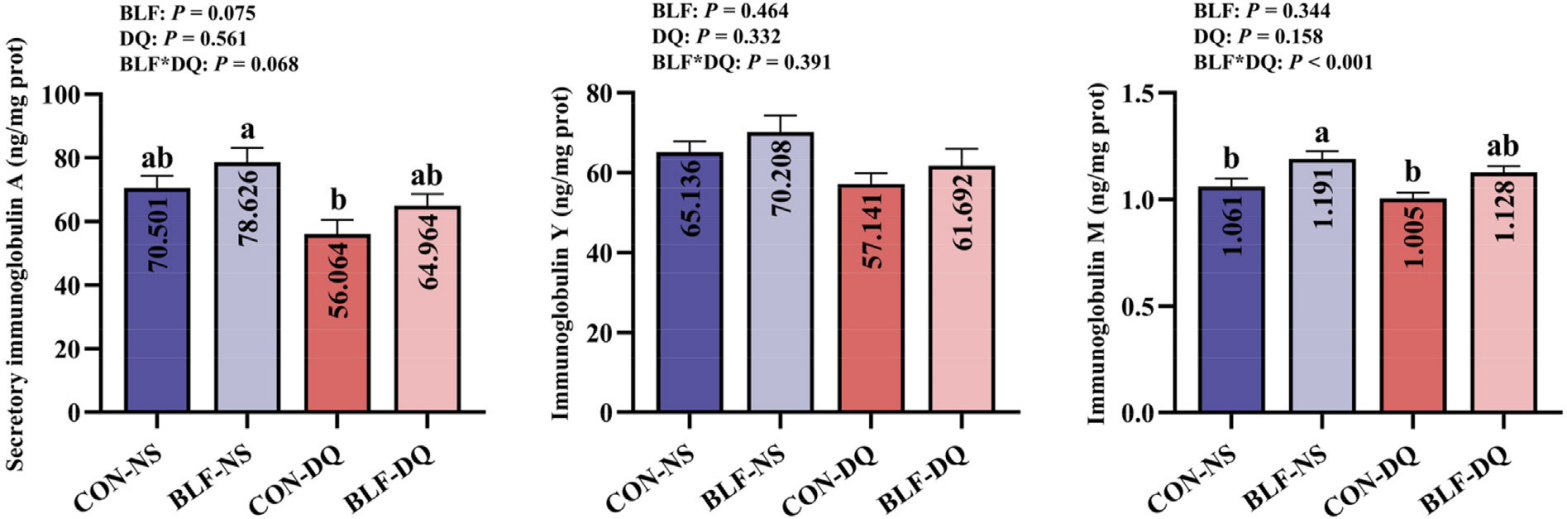
Effects of the bamboo leaf flavonoids (BLF) on jejunal immunoglobulins in diquat (DQ) stressed broilers. Columns labeled with different letters indicate significant differences (*P* < 0.05), while with no letter or the same letter superscripts mean no significant difference (*P* > 0.05). *n* = 8.

Adding BLF to the feed increased the serum IgM level (*P* = 0.019; Fig. 4). Diquat challenge decreased the IgY (*P* = 0.036) and IgM (*P* = 0.032) concentrations. Results showed a significant interaction between BLF and DQ in IgA level (*P* = 0.002). Compared with CON-NS group, BLF-NS group increased serum IgA and IgM levels (*P* < 0.05). Compared with CON-DQ group, BLF-DQ group increased serum IgA and IgM levels (*P* < 0.05). No significant discrepancy between CON-NS group and BLF-DQ group in the contents of serum IgA, IgM and IgY in broilers were shown (*P* > 0.05). It presented a significant interaction between BLF and DQ in jejunal mucosa IgM levels (*P* < 0.001; Fig. 5). The content of jejunal mucosa IgM in BLF-NS group was higher than that in CON-NS group (*P* < 0.05).

### 3.5 Serum and jejunal mucosa cytokines

**Fig. 6:**
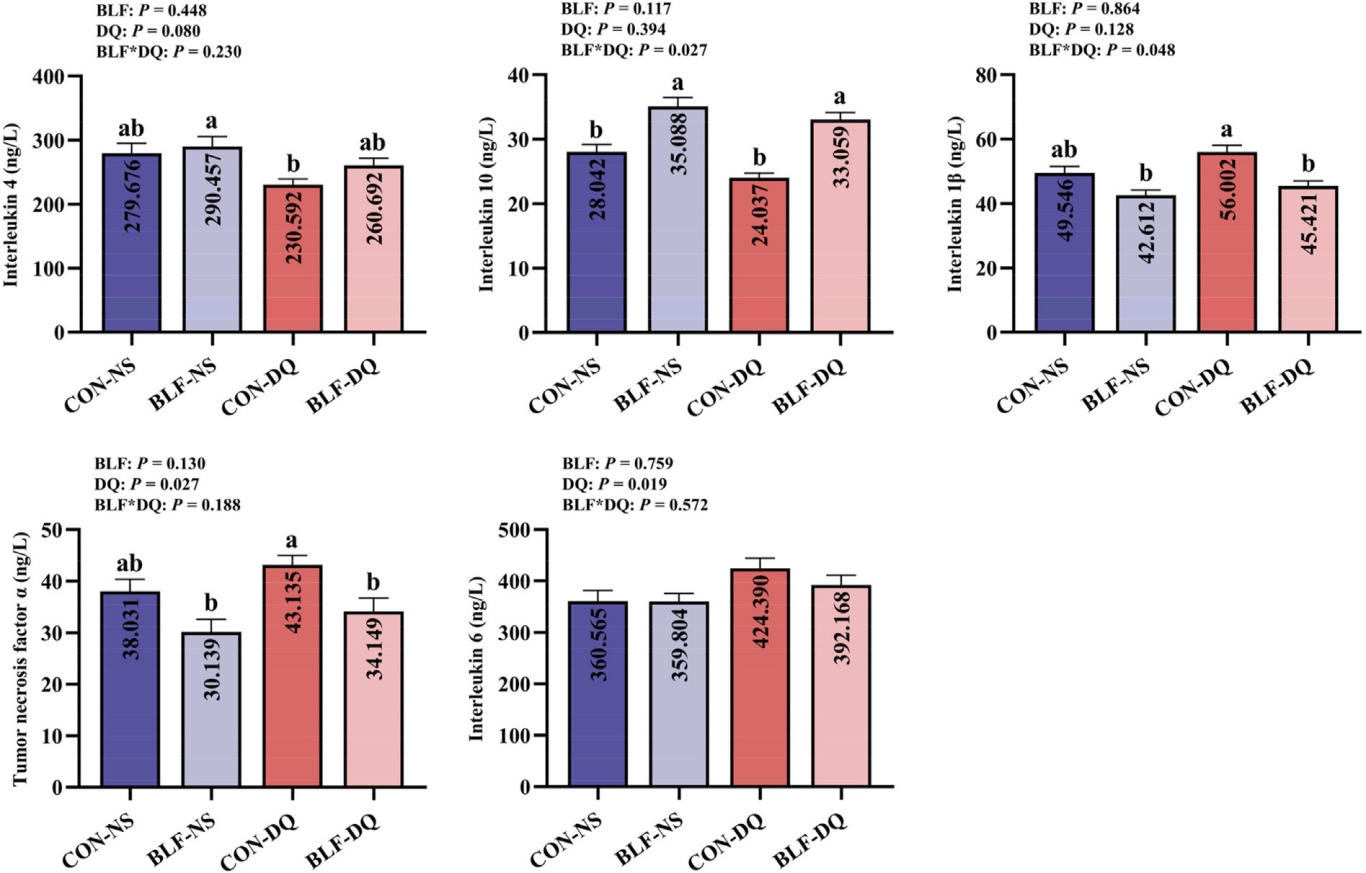
Effects of the bamboo leaf flavonoids (BLF) on serum cytokines in diquat (DQ) stressed broilers. Columns labeled with different letters indicate significant differences (*P* < 0.05), while with no letter or the same letter superscripts mean no significant difference (*P* > 0.05). *n* = 8.

Effects of the BLF on serum cytokines in broilers under DQ stress are shown in Fig. 6. Diquat stress led to an increase in the levels of TNF-α (*P* = 0.027) and IL-6 (*P* = 0.019). There was a significant interaction between BLF and DQ on serum IL-10 (*P* = 0.027) and IL-1β (*P* = 0.048) levels. Compared with CON-NS group, the concentration of IL-10 in BLF-NS group was improved (*P* < 0.05). Compared with CON-DQ group, the serum IL-10 level increased, the contents of IL-1β and TNF-α in BLF-DQ group decreased (*P* < 0.05). There were no significant differences in the contents of IL-4, IL-1β, TNF-α and IL-6 between CON-NS group and BLF-DQ group (*P* > 0.05).

**Fig. 7:**
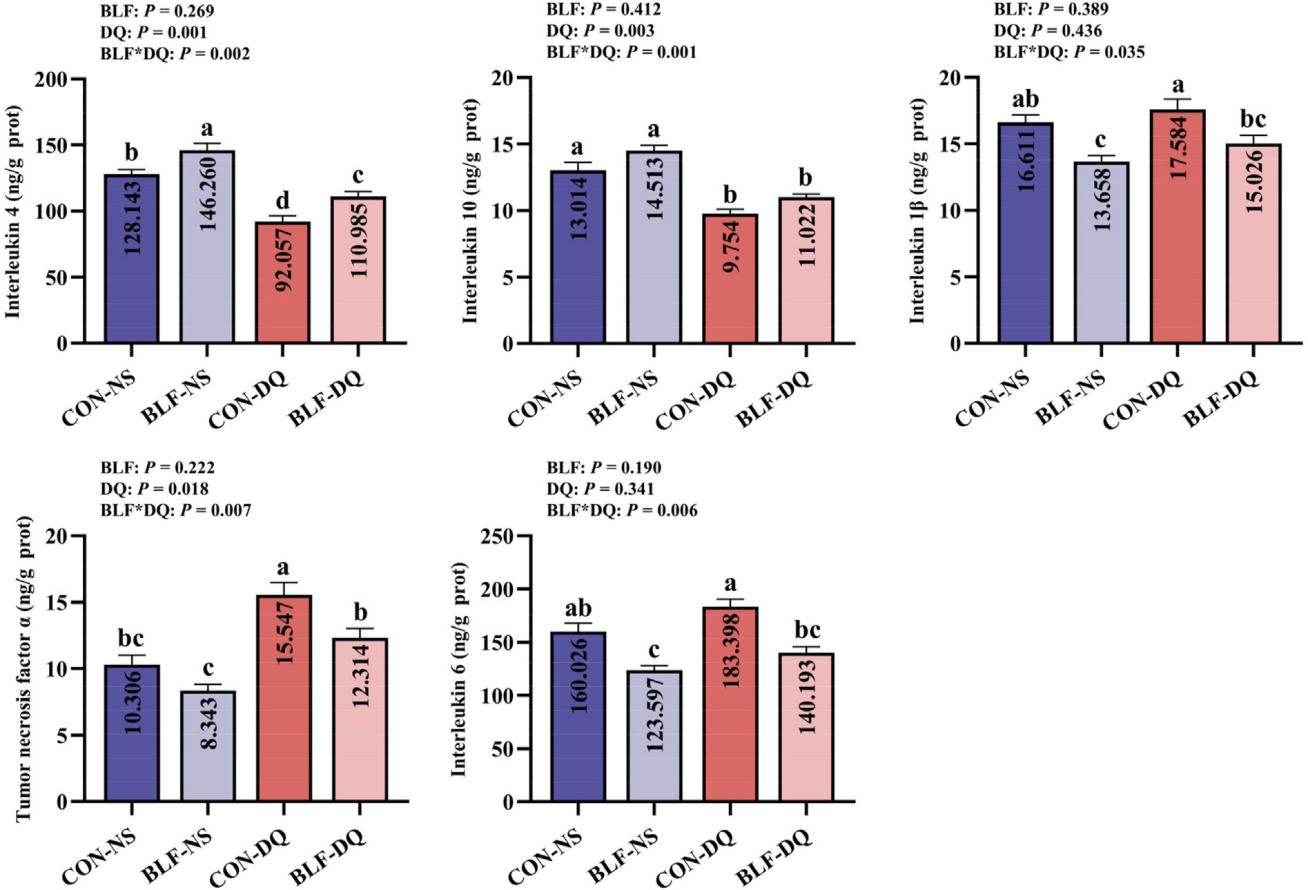
Effects of the bamboo leaf flavonoids (BLF) on jejunal mucosa cytokines in diquat (DQ) stressed broilers. Columns labeled with different letters indicate significant differences (*P* < 0.05), while with no letter or the same letter superscripts mean no significant difference (*P* > 0.05). *n* = 8.

Diquat challenge reduced the jejunal mucosa IL-4 (*P* = 0.001) and IL-10 (*P* = 0.003) concentration (Fig. 7), increased the TNF-α concentration (*P* = 0.018). There were interactions between BLF addition and DQ stress on jejunal mucosa IL-4 (*P* = 0.002), IL-10 (*P* = 0.001), IL-1β (*P* = 0.035), TNF-α (*P* = 0.007), and IL-6 (*P* = 0.006) concentrations. In comparison to CON-NS group, adding BLF to the diet significantly enhanced the content of jejunal mucosa IL-4 (*P* < 0.05), diminished the contents of IL-1β and IL-6 (*P* < 0.05). Under DQ stress, adding BLF to diet improved IL-4 content (*P* < 0.05), and reduced IL-1β, TNF-α and IL-6 contents (*P* < 0.05).

### 3.6 Morphology of jejunal mucosa tissue

**Fig. 8:**
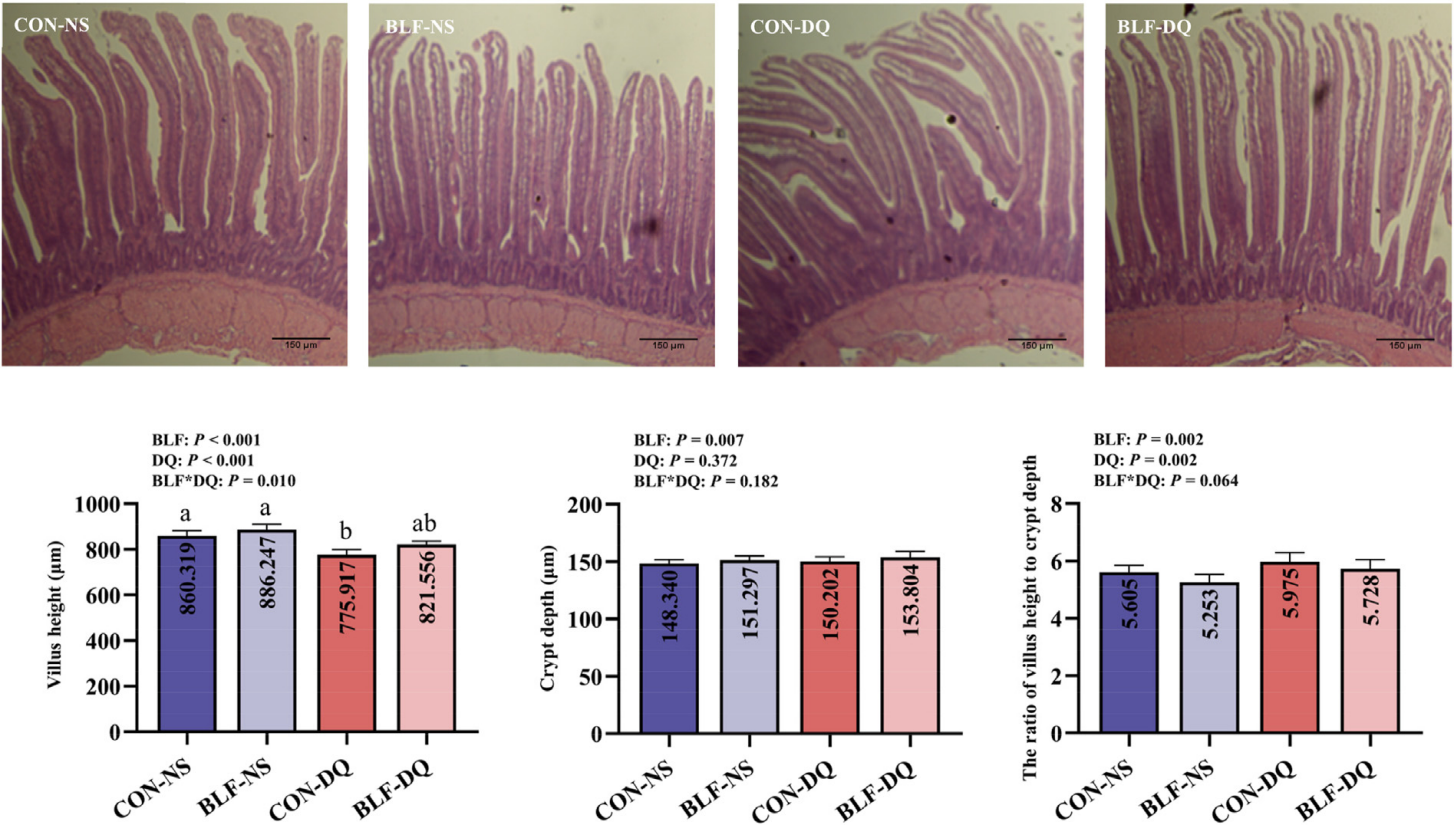
Effects of the bamboo leaf flavonoids (BLF) on jejunal mucosa morphology in diquat (DQ) stressed broilers. Columns labeled with different letters indicate significant differences (*P* < 0.05), while with no letter or the same letter superscripts mean no significant difference (*P* > 0.05). *n* = 8.

Dietary supplementation with BLF has significant impacts on villus height (*P* < 0.001), crypt depth (*P* = 0.007) and the ratio of villus height to crypt depth (*P* = 0.002; Fig. 8). There was a significant interaction between BLF addition and DQ stress on villus height (*P* = 0.010). And result showed no significant differences in villus height, crypt depth and the ratio of villus height to crypt depth between CON-NS group and BLF-DQ group (*P* > 0.05).

### 3.7 Expressions of jejunal mucosa barrier related genes

**Fig. 9:**
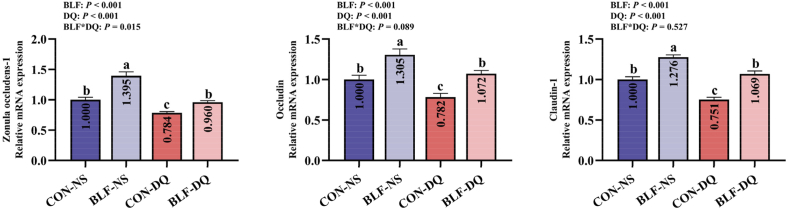
Effects of the bamboo leaf flavonoids (BLF) to jejunal mucosa barrier on expression of genes related in diquat (DQ) stressed broilers. Columns labeled with different letters indicate significant differences (*P* < 0.05), while with no letter or the same letter superscripts mean no significant difference (*P* > 0.05). *n* = 8.

Adding BLF to the feed increased the genes expression levels of *z*onula occludens-1 (*ZO-1*, *P* < 0.001), occludin (*OCLN*, *P* < 0.001) and claudin-1 (*CLDN1*, *P* < 0.001; Fig. 9). Diquat challenge reduced the expression of *ZO-1* (*P* < 0.001), *OCLN* (*P* < 0.001) and *CLDN1* (*P* < 0.001). There was a significant interaction between BLF and DQ in *ZO-1* expression (*P* = 0.015). In comparison to CON-DQ group, the relative expression of *ZO-1*, *OCLN* and *CLDN1* in BLF-DQ group was overexpressed (*P* < 0.05). There were no significant differences between CON-NS group and BLF-DQ group on relative expression of *ZO-1*, *OCLN* and *CLDN1* genes (*P* > 0.05).

### 3.8 Volatile fatty acids in cecal contents

**Fig. 10:**
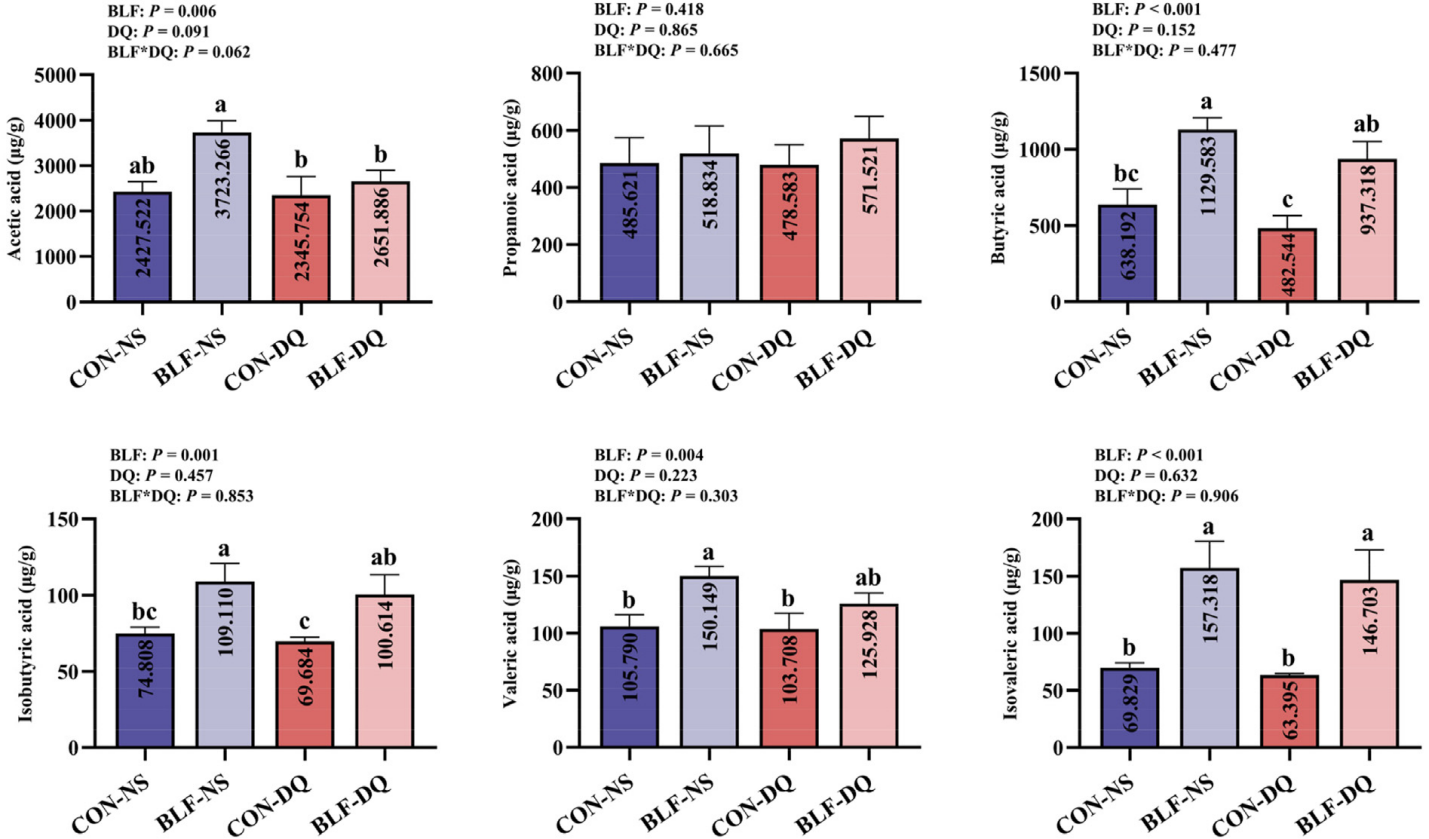
Effects of the bamboo leaf flavonoids (BLF) on volatile fatty acid in diquat (DQ) stressed broilers. Columns labeled with different letters indicate significant differences (*P* < 0.05), while with no letter or the same letter superscripts mean no significant difference (*P* > 0.05). *n* = 8.

The addition of BLF in the diet increased the contents of cecal volatile fatty acids, including acetic acid (*P* = 0.006), butyric acid (*P* < 0.001), isobutyric acid (*P* = 0.001), valeric acid (*P* = 0.004) and isovaleric acid (*P* < 0.001; Fig. 10). Compared to CON-NS group, there were elevated levels of butyric acid, valeric acid, isobutyric acid and isovaleric acid of broilers in BLF-NS group (*P* < 0.05). In comparison to CON-DQ group, the contents of butyric acid, isobutyric acid and isovaleric acid in BLF-DQ group were increased (*P* < 0.05). Compared with CON-NS group, the levels of acetic acid, propanoic acid, butyric acid, isobutyric acid and valeric acid in BLF-DQ group had no significant differences (*P* > 0.05), while the level of isovaleric acid was increased (*P* < 0.05).

### 3.9 Microbial flora of cecal contents

**Fig. 11:**
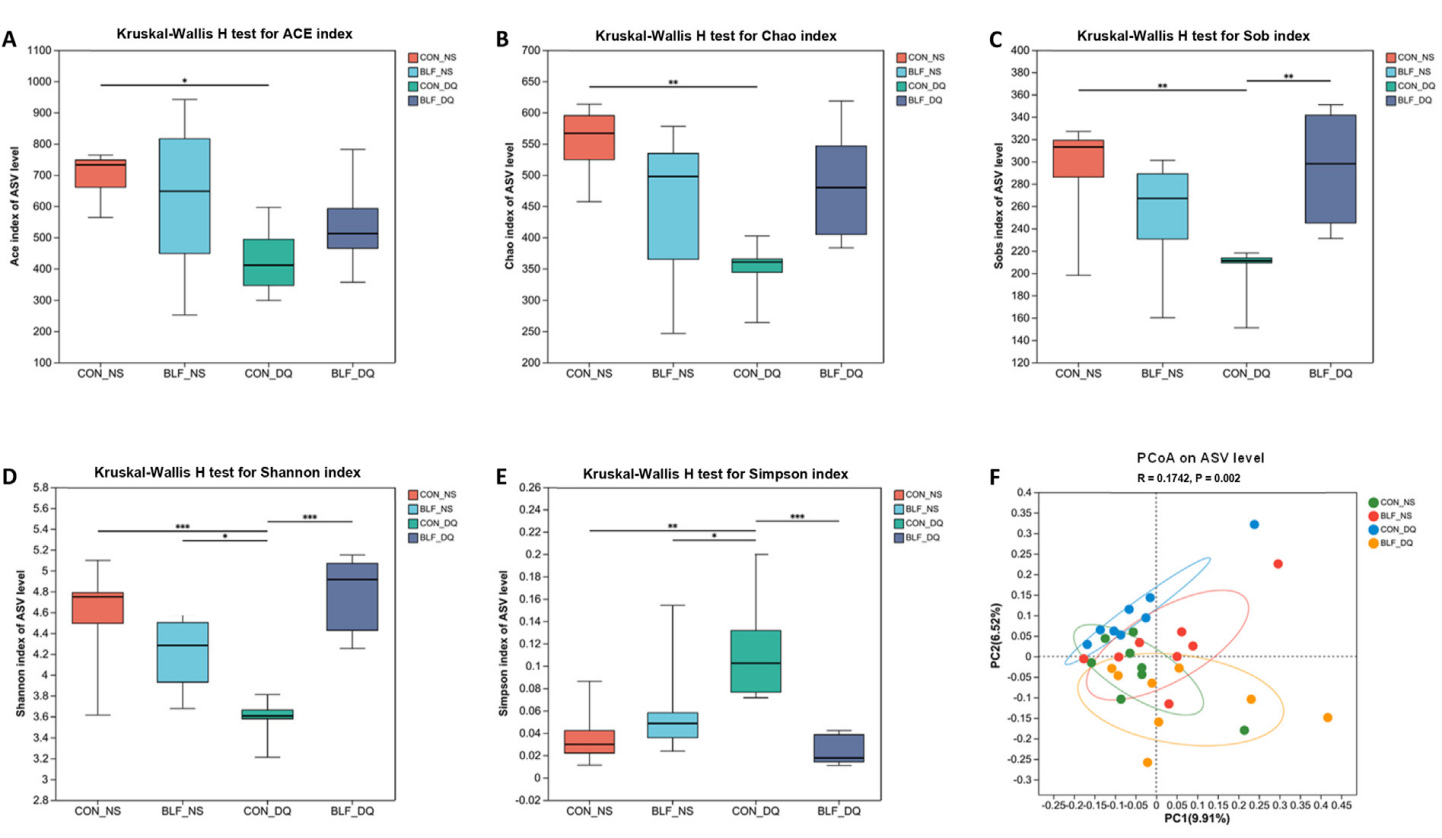
Effects of bamboo leaf flavonoids (BLF) on species diversity of cecal microbiota in diquat (DQ) stress broilers. Asterisks (∗) indicate significant difference (*P* < 0.05). Double asterisks (∗∗) indicate significant difference (*P* < 0.01). Triple asterisks (∗∗∗) indicate significant difference (*P* < 0.001). (A to F) Kruskal–Wallis H test for abundance-based coverage (ACE) index, Chao index, the observed species index (Sobs index), Shannon index and Simpson index, respectively. (F) PCoA on ASV level. PCoA = principal co-ordinates analysis; ASV = amplicon sequence variant.

As shown in Fig. 11, compared with CON-NS group, DQ stress reduced the Ace, Chao and Sobs indices of cecal microbiota (*P* < 0.05). Compared with CON-DQ group, the Sobs index of cecal microbiota in the BLF-DQ group was increased (*P* < 0.05). There was no noticeable difference between BLF-DQ group and CON-NS group in Ace, Chao and Sobs indices (*P* > 0.05). Compared with CON-NS group, CON-DQ group had a significant decrease in Shannon index (*P* < 0.05) while a significant increase in Simpson index (*P* < 0.05). Compared with CON-DQ group, the addition of BLF increased Shannon index and reduced Simpson index (*P* < 0.05). Based on the representation of PCoA β-diversity analysis, remarkable discrepancies in species composition were observed between the CON-NS group and the CON-DQ group, with BLF-NS and BLF-DQ groups in between and closer to the CON-NS group. ANOSIM similarity analysis showed R = 0.1742 and *P* = 0.002, indicating that inter-group differences were greater than intra-group differences.

**Fig. 12:**
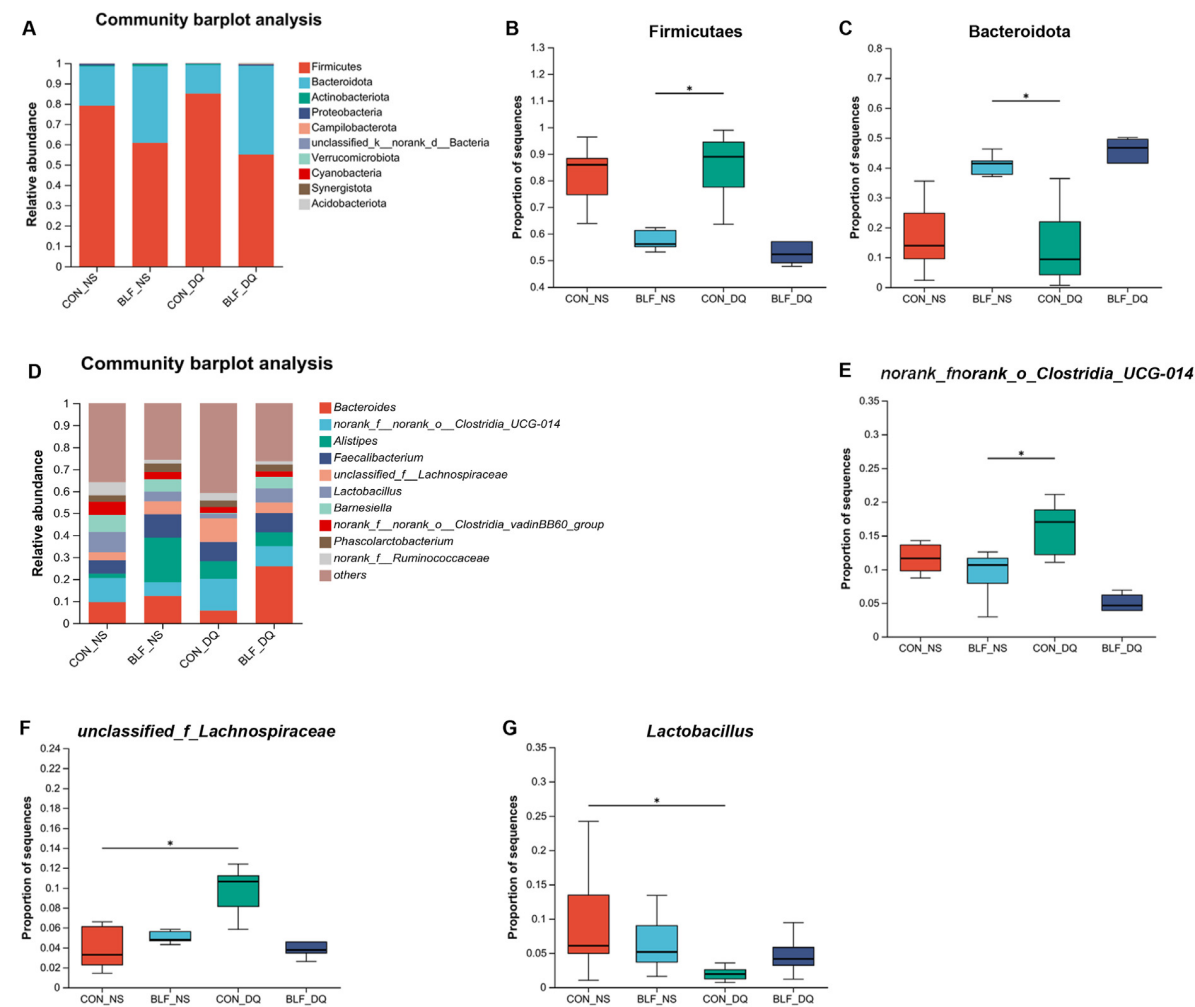
Effects of bamboo leaf flavonoids (BLF) on cecal microflora diversity in diquat (DQ) stressed broilers. Asterisks (∗) indicate significant difference (*P* < 0.05). (A) Community barplot analysis (Phylum). (B) Firmicutes. (C) Bacteroidota. (D) Community barplot analysis (Genus). (E) *norank_fnorank_o_Clostridia_UCG-014*. (F) *unclassified_f_Lachnospiraceae*. (G) *Lactobacillus*.

At the phylum level, Firmicutes, Bacteroidota, Actinobacteriota, Proteobacteria and Campilobacterota emerged as the most predominant taxa in the cecal microbiota of broilers, as illustrated in Fig. 12. Compared with CON-DQ group, the supplement of BLF reduced the relative abundance of cecum *Firmicutes* of broilers (*P* < 0.05), while boosting the relative abundance of Bacteroidota (*P* < 0.05). At the cecum genus level, the microbiota was mainly composed of *Bacteroides*, *norank_f_norank_o_Clostridia_UCG-014*, *Alistipes* and *unclassified_f_Lachnospiraceae.* The relative abundance of *norank_f_norank_o_Costridia_UCG-014* in the cecum in BLF-DQ group was lower than that in CON-DQ group (*P* < 0.05). Compared with CON-NS group, CON-DQ group showed an increase in the relative abundance of *unclassified_f_Lachnospiraceae* (*P* < 0.05) and a decrease in the relative abundance of *Lactobacillus* in broilers (*P* < 0.05).

## 4 Discussion

Studies have shown that adding plant extracts rich in flavonoids to feed could improve the growth performance of broilers (Chen et al., 2020a; Ouyang et al., 2013). The administration of BLF significantly increased the average daily weight gain, average daily feed intake and decreased the F/G of broilers (Cao et al., 2023). In the current work, the addition of BLF significantly decreased the F/G of broilers in the early stage, which was consistent with previous research findings that adding flavonoids to feed could substantially reduce the F/G of yellow feathered broilers (Xue et al., 2021). Research has found that flavonoids could promote the proliferation of small intestinal epithelial cells and improve intestinal morphology (Teng et al., 2023). Therefore, adding BLF to the diet reduced F/G may be due to improving the ability of intestinal development, promoting nutrient absorption, and thereby enhancing the growth performance of broilers.

Oxidative damage is a loss of balance between free radical levels and cellular antioxidant capacity, resulting in relatively insufficient cellular antioxidant capacity and increased ROS, leading to cellular and tissue damage (Schieber and Chandel, 2014). In this experiment, the serum and jejunal mucosa ROS levels of broilers in DQ group significantly increased than normal levels, indicating that excessive free radicals were produced after DQ stress. Additionally, in comparison to the CON-DQ group, the serum ROS content in BLF-DQ significantly decreased. This indicates that BLF was able to reduce high ROS production and regulate the related antioxidant defense response. No significant differences in the levels of T-AOC, CAT, GPx, T-SOD, MDA between CON-NS group and BLF-DQ group of broilers were revealed. This may be related to the fact that flavonoids have the ability to transfer electron free radicals, chelate metal catalysts, activate antioxidant enzymes (Parcheta et al., 2021). Therefore, BLF should have a certain protective effect on broilers in coping with oxidative stress. A previous study has proved that there was a linear increase in T-AOC activity of broilers with the increase in the dose of bamboo leaf extract rich in flavonoids (Shen et al., 2019b). Other research has demonstrated that increasing the expressions of gene *SOD*, *GPx*, and *CAT* could reduce lipid oxidation so as to improve the antioxidant function in broilers (Shen et al., 2019a). Data in the present study were in accordance with these results, in which BLF could increase the serum T-AOC and CAT, jejunal T-AOC and T-SOD activities and reduce MDA content in DQ stressed broilers. It is possible that the dietary BLF act as natural antioxidant and improve the ability to cope with oxidative stress by enhancing relevant enzyme antioxidant system.

The main site for digesting feed and absorbing nutrients is the jejunum (Takizawa et al., 2020). Maintaining a healthy state of jejunal barrier function is the foundation for normal growth (Hu et al., 2024). The contents of serum D-LA and DAO usually reflect the extent of injury to intestinal barrier function and permeability (Chen et al., 2015). It was reported that the excessive elevation of D-LA and DAO could be reversed by flavonoids (Nong et al., 2024). In this study, DQ stress resulted in abnormally elevated D-LA and DAO levels. BLF significantly reduced DAO concentration of DQ stressed broilers, indicating that BLF were involved in repairing oxidative stress damage in the intestines of broilers and had a protective impact on the intestinal mucosa. Intestinal tight junction proteins are important structural basis for maintaining intestinal epithelial cell barrier (Liang et al., 2019). The present study not only found that BLF supplementation increased the expressions of *CLDN1*, *ZO-1* and *OCLN*, but also revealed that BLF restored those genes in DQ stressed broilers to normal levels. This was consistent with previous research findings indicating that supplementing flavonoids in diet could upgrade the *CLDN1* and *ZO-1* expressions in broilers (Zhang et al., 2023). The above results suggested that BLF could relieve DQ stress induced intestinal damage, this may be related to the excellent antioxidant, antibacterial and anti-inflammatory effects of flavonoids (Zhang et al., 2017).

Immunoglobulins and inflammatory factors have a significant effect on immune function and anti-infection response (Bai et al., 2017). Immunoglobulin A is related to mucosal immunity, IgM is associated with acute infection and IgY is a key circulating antibody in birds (Li et al., 2020; Pacheco et al., 2023). It has been confirmed that TNF-α, anti-inflammatory cytokines, pro-inflammatory cytokines have salient effects in regulating inflammation, immune defense tissue development, and lymphocyte homeostasis (Croft et al., 2013; Volpin et al., 2014). Bamboo leaf flavonoids have direct anti-inflammatory effects on macrophages and could resist pro-inflammatory genes and inflammatory genes expressions (Yang et al., 2017). Similarly, this experiment discovered that BLF contributed to regulating the secretion of immunoglobulins and anti-inflammatory factors. In the absence of oxidative stress in broilers, adding BLF significantly improved the contents of serum IgA, IgM, IL-10 and jejunal IgM, IL-4, while decreasing content of IL-6. When compared to CON-DQ, the supplemental BLF raised the serum IgA, IgM, IL-10 levels and reduced IL-1 β and TNF-α contents, increased jejunum IL-4 content and diminished jejunum IL-1β, TNF-α and IL-6 contents. Therefore, it was speculated that BLF could enhance the immunity and have a positive impact on intestinal immune barrier function of broilers.

As a protective barrier, the intestinal epithelium is used to assess the integrity of intestinal development and function, playing an active role in nutrient absorption (Farré et al., 2020). The microstructure of the small intestine is considered to be a major indicator of intestinal development, health, and function (Yaqoob et al., 2021). The intestinal mucosa contains high concentrations of non-protein sulfhydryl groups (Murakami et al., 1988). Excessive ROS acts on the sulfhydryl group, denaturing the protein and inactivating the enzyme, causing lipid peroxidation damage, damaging the intestinal mucosa, thinning the intestinal mucus layer, shortening the villi, and shallowing the crypt (Wang et al., 2021a). This result discovered that DQ stress reduced the villus height and the ratio of villus height to crypt depth in broilers. The current study showed that diet supplemented with BLF significantly elevated broilers jejunum villus height and the ratio of villus height to crypt depth. The results were similar to the previous studies that flavonoids enhanced small intestine mucosal integrity by reducing crypt depth in broilers (Gao et al., 2024). Pathogenic bacteria are the main cause of gastrointestinal diseases, which may damage intestinal villi and have adverse effects on the digestion and absorption of nutrients (Adedokun and Olojede, 2019). Bamboo leaf flavonoids have noteworthy antibacterial properties and could effectively combat various pathogenic bacteria in the digestive tract, with the most significant antibacterial effect on *Escherichia coli* (Noremylia et al., 2023). Accordingly, the improvement of intestinal morphology may be associated with the antioxidant and antibacterial effects of BLF.

Volatile fatty acids are conducive to maintain the normal pH value of intestine, restrain the survival of pernicious bacteria and supply energy for intestinal epithelial cells (Li et al., 2023). Citrus peel flavonoid extract could promote the production of acetic acid, which is beneficial for maintaining intestinal homeostasis (Li et al., 2022). The present results disclosed that adding BLF in feed increased cecal contents of acetic acid, propionic acid, isobutyric acid and valeric acid of broilers. Moreover, broilers in BLF-DQ group had a higher content of isovaleric acid when compared to CON-NS group. This was consistent with the role of BLF in improving intestinal morphology in this study. These suggested that BLF may improve intestinal development by regulating the content of VFA.

There is a symbiosis between the intestinal microbiota and animals, and the intestinal microbiota could regulate the levels of metabolites in the serum, alter the host’s immune function and intestinal permeability (Dodd et al., 2017). Microbiota richness (Ace, sobs and Chao indices) and microbial diversity (Shannon and Simpson indices) can be used as indicators to measure the stability and resilience of intestinal microorganisms (Chen et al., 2020b). This study found DQ stress declined the cecal microbiota Ace, Chao, Sobs and Shannon indices, indicating that the cecum microbial richness and diversity in DQ stressed broilers was reduced, while the addition of BLF significantly increased the Sobs index. However, differences between BLF-DQ group and CON-NS group in cecal microbial flora indicated no significance. The beneficial effects of natural flavonoids on regulating the gut microbiota of poultry have been reported (Zhang et al., 2023). In the current study, it was indicated that BLF was helpful to mitigate the negative impacts of DQ challenge on intestinal microbiota. The cecal flora of broilers is dominated by Firmicutes and Bacteroides (Fan and Pedersen, 2021). It has been discovered that broilers with improved growth performance had more relative richness of Bacteroidetes and less relative richness of Firmicutes (Li et al., 2016). Consistently, the present result showed that BLF significantly decreased the relative abundance of Firmicutes and improved the relative abundance of Bacteroides on DQ stressed broilers. Thus, the addition of flavonoids to diet might promote growth performance by regulating intestinal flora.

*Lactobacillus* is a common intestinal probiotic that can secrete various antibacterial substances and mucus, enhance the immune defense ability of the intestinal barrier, prevent the invasion of harmful substances, and promote the proliferation of other microorganisms (Zhang et al., 2023). At the genus level, this result demonstrates that *Lactobacillus* relative abundance significantly decreased in broilers under DQ stress, posing a threat to the intestinal health of broilers. Additionally, there was no significant difference on the abundance of *Lactobacillus* between BLF-DQ group and CON-NS group. It indicated that the addition of BLF to feed was beneficial for mitigating the detrimental effect of DQ stress on *Lactobacillus*. It was reported that oral administration of flavonoid aminoglycosides to tumor bearing mice significantly raised the abundance of *Lactobacillus* (Chen et al., 2021a). Additionally, flavonoids improved CTX induced immune dysfunction in mice, while significantly upregulating the relative abundance of *norank_f_norank_o_Clostridia_UCG-014*, indicating that *norank_f_norank_o_Clostridia_UCG-014* may be effective in improving immune function (Xiao et al., 2023). In this study, BLF increased the relative abundance of n *norank_f_norank_o_Clostridia_UCG-014* in the cecum of DQ stressed broilers. Therefore, it was speculated that BLF could improve the immune system of broilers by regulating the abundance of *Lactobacillus* and *norank_f_norank_o_Clostridia_UCG-014* in the cecum.

## 5 Conclusion

In summary, bamboo leaf flavonoids could improve the feed utilization of broilers. By establishing a diquat stress injury model, it has been elucidated that bamboo leaf flavonoids may enhance antioxidant function, strengthen immune system, regulate volatile fatty acid contents, improve intestinal morphology and microbiota and other intestinal barrier functions, so as to mitigate the injury induced by oxidative stress in broilers. The current research findings offer a practical strategy for the repair of oxidative damage in broilers.

## CRediT authorship contribution statement

**Qilu Zhou:** Writing – original draft, Formal analysis. **Sikandar Ali:** Resources, Data curation. **Xueyan Shi:** Validation. **Guangtian Cao:** Validation. **Jie Feng:** Supervision. **Caimei Yang:** Funding acquisition, Conceptualization. **Ruiqiang Zhang:** Writing – review & editing, Project administration, Methodology, Conceptualization.

## Declaration of competing interest

We declare that we have no financial and personal relationships with other people or organizations that can inappropriately influence our work, and there is no professional or other personal interest of any nature or kind in any product, service and/or company that could be construed as influencing the content of this paper.
